# Mechanistic Insight of Bivalent Compound 21MO as Potential Neuroprotectant for Alzheimer’s Disease

**DOI:** 10.3390/molecules21040412

**Published:** 2016-03-25

**Authors:** John M. Saathoff, Kai Liu, Jeremy E. Chojnacki, Liu He, Qun Chen, Edward J. Lesnefsky, Shijun Zhang

**Affiliations:** 1 Department of Medicinal Chemistry, Virginia Commonwealth University, Richmond, VA 23298, USA; saathoffjm@vcu.edu (J.M.S.); kliu4@vcu.edu (K.L.); chojnackij@vcu.edu (J.E.C.); lhe3@vcu.edu (L.H.); 2 Department of Medicine, Pauley Heart Center, Division of Cardiology, Virginia Commonwealth University, Richmond, VA 23298, USA; qchen8@vcu.edu (Q.C.); ejlesnefsky@vcu.edu (E.J.L.); 3 Department of Biochemistry, Virginia Commonwealth University, Richmond, VA 23298, USA

**Keywords:** Alzheimer’s disease, bivalent compound, calcium, mitochondria, multifunctional, neuroprotection

## Abstract

We have recently developed a bivalent strategy to provide novel compounds that potentially target multiple risk factors involved in the development of Alzheimer’s disease (AD). Our previous studies employing a bivalent compound with a shorter spacer (**17MN**) implicated that this compound can localize into mitochondria and endoplasmic reticulum (ER), thus interfering with the change of mitochondria membrane potential (MMP) and Ca^2+^ levels in MC65 cells upon removal of tetracycline (TC). In this report, we examined the effects by a bivalent compound with a longer spacer (**21MO**) in MC65 cells. Our results demonstrated that **21MO** suppressed the change of MMP, possibly via interaction with the mitochondrial complex I in MC65 cells. Interestingly, **21MO** did not show any effects on the Ca^2+^ level upon TC removal in MC65 cells. Our previous studies suggested that the mobilization of Ca^2+^ in MC65 cells, upon withdraw of TC, originated from ER, so the results implicated that **21MO** may preferentially interact with mitochondria in MC65 cells under the current experimental conditions. Collectively, the results suggest that bivalent compounds with varied spacer length and cell membrane anchor moiety may exhibit neuroprotective activities via different mechanisms of action.

## 1. Introduction

Alzheimer’s disease (AD) is a devastating neurodegenerative disorder and the most common cause of dementia [1]. The complexity of this disease makes drug development efforts to provide effective disease modifying agents a challenging and unmet task since multiple pathogenic factors have been implicated in the development of AD, such as amyloid-β (Aβ) aggregates [2,3,4,5], oxidative stress, neuroinflammation, and mitochondria dysfunction, among others [6,7,8]. To address this challenge, the multifunctional strategy of small molecule design by employing molecular conjugation or hybridization has recently attracted extensive attention in surmounting the paucity of effective disease-modifying agents in the pipeline of AD therapeutics [9,10,11].

**Figure 1:**
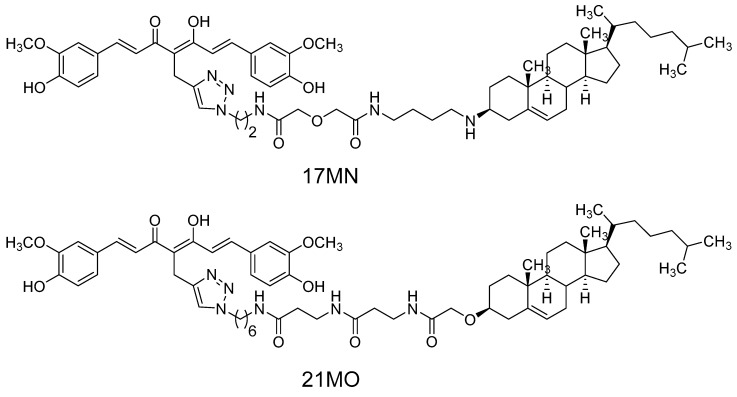
Chemical structures of **17MN** and **21MO**.

Recently, we developed a novel bivalent ligand strategy to link a multifunctional “war head”, namely curcumin, with a cell membrane/lipid raft (CM/LR) anchor moiety into our molecular design [12,13,14]. Our results demonstrated that this bivalent strategy provided compounds with significantly improved neuroprotection compared to either curcumin or the CM/LR anchor alone, or the combination of these two [12,13,14]. The spacer length between the “war head” and the anchorage moiety proved to be critical for their neuroprotections. Further mechanistic studies employing one of these lead bivalent compounds as a probe (**17MN**, Figure 1) demonstrated that our bivalent compound can reverse the change of mitochondrial membrane potential (MMP) and cytosolic Ca^2+^ levels induced by the withdrawal of tetracycline (TC) in our MC65 cell model system, thus protecting MC65 cells from TC-removal induced necroptosis [15]. MC65 is a human neuroblastoma cell line that conditionally expresses a 99-residue carboxyl terminal fragment of Aβ precursor protein (APP) and Aβ after removal of TC. This cell line is widely recognized as one of the cellular models of AD, resulting in intracellular Aβ oligomers (AβOs) formation and oxidative stress. Furthermore, our studies indicated that **17MN** interacts with both mitochondria and endoplasmic reticulum (ER), thus suggesting a multiple-site mechanism for the observed neuroprotective activities in MC65 cells for our bivalent compounds. Our studies also noticed that bivalent compounds with varied spacer lengths exhibit differential neuroprotection profile in MC65 cells [12,13,14,15]. Therefore, it would be interesting to examine how these bivalent compounds with varied spacer lengths behave differentially in the cellular model system. Herein, we report the characterization of another lead bivalent compound, **21MO** (Figure 1), with a longer spacer, in MC65 cells and compare its effects on MMP and Ca^2+^ change to our previously reported bivalent compound **17MN**.

## 2. Results and Discussion

**Figure 2:**
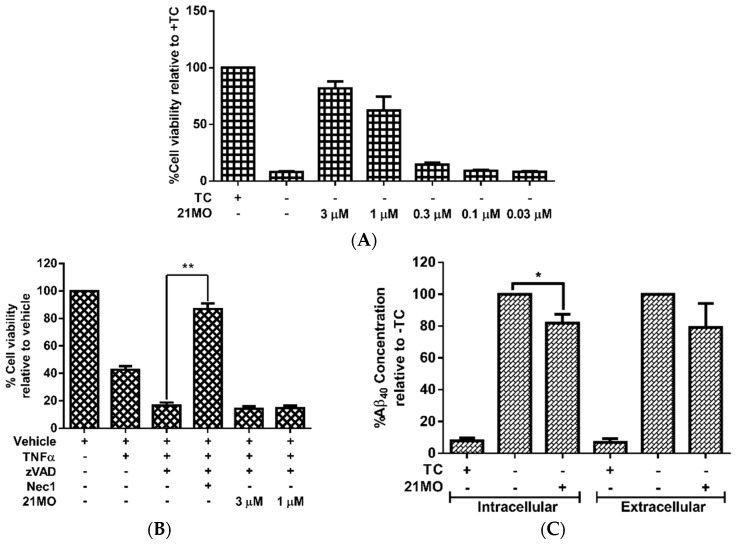
The influence of **21MO** on the necroptosis and total Aβ_40_. (**A**) MC65 cells were treated with **21MO** (1 µM) under −TC conditions for 72 h. Cell viability was assessed by MTT (3-(4,5-dimethylthiazol-2-yl)-2,5-diphenyltetrazolium bromide) assay; (**B**) U937 cells were pretreated with **21MO** (1 or 3 µM) or Necrostatin-1 (10 µM) and caspase pan inhibitor zVAD (10 µM) for 1 h, then TNF-α (50 ng/mL) was added and incubated for 72 h. Cell viability was assessed by MTT assay. (** *p* < 0.01); (**C**) MC65 cells were treated with **21MO** (1 µM) under −TC conditions for 48 h. Medium was collected for analysis of extracellular Aβ_40_. Cells were lysed and analyzed for intracellular Aβ_40_. Total Aβ_40_ concentrations were normalized by total protein content of lysed cells. (* *p* < 0.05).

Our previous studies have shown that, upon removal of TC, MC65 cells die through necroptosis and bivalent compounds, **17MN** protects MC65 cells from TC-removal induced cytotoxicity by engaging target proteins between receptor interacting protein kinase-1 (RIPK1) and Aβ [15]. Therefore, we initially tested **21MO** in MC65 cells to compare whether it functions similarly to **17MN**. The results demonstrated that **21MO** can efficiently rescue MC65 cells from TC-removal induced necroptosis (Figure 2A) and but could not rescue TNF/zVAD induced necroptosis in U937 cells (Figure 2B), thus suggesting that **17MN** and **21MO** may function similarly in MC65 cells under the current experimental conditions. Further studies also demonstrated that **21MO** (1 μM) slightly decreased the intracellular level of Aβ_40_, but did not interfere with the level of extracellular Aβ_40_ (Figure 2C). Overall, **21MO** did not significantly inhibit the total production of Aβ_40_, especially when comparing the reduction of intracellular Aβ_40_ with the neuroprotective activity of **21MO** at this concentration. Our previous studies demonstrated that **21MO** exhibits inhibitory effects on the aggregation of small AβOs, but with a much weaker potency compared to its inhibition of MC65 cell death [12], thus suggesting that the inhibition of Aβ aggregation might only contribute partially towards its overall neuroprotective ability. Taken together, the results suggest that other proteins are involved in the observed neuroprotective activities by these bivalent compounds in MC65 cells.

**Figure 3:**
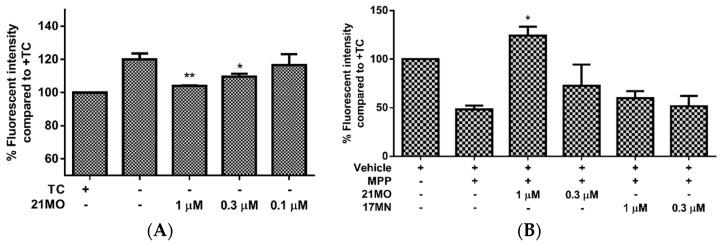
The influence of **21MO** on mitochondrial potential. (**A**) MC65 cells were treated with **21MO** at indicated concentrations under −TC condition for 48 h. Cells were then incubated with TMRM (100 nM) for 30 min. Mean fluorescence intensity was measured by flow cytometry. (* *p* < 0.05; ** *p* < 0.01 compared to −TC control); (**B**) SH-SY5Y cells were treated with either **21MO** or **17MN** at indicated concentrations and MPP^+^ (2.5 mM) for 24 h. Cells were then incubated with TMRM (100 nM) for 30 min. Mean fluorescence intensity was measured by flow cytometry. (* *p* < 0.05 compared to MPP treated group).

Next, we set out to examine the effects of **21MO** on the change of MMP upon the removal of TC in MC65 cells in comparison with **17MN**. Consistent with our previous results, the MC65 cells are hyperpolarized (increase in MMP) after the removal of TC and this may indicate the interactions of AβOs with mitochondria caused the change of MMP. Notably, **21MO** suppressed the increase of MMP at 0.3 and 1 µM concentrations (Figure 3A). This may suggest that our bivalent compounds, regardless of spacer lengths, interact with mitochondria to interfere with the effects of AβOs on the mitochondria. Our recent studies have shown that reactive oxygen species (ROS), especially mitochondrial ROS (mitoROS), are involved in the cell death of MC65 cells [13,16]. Furthermore, complex I of the electron transport chain is associated with the production of mitoROS [17]. Then, another cellular model employing neuroblastoma SH-SY5Y cells in the presence of MPP^+^, a neurotoxin targeting the complex I of mitochondria, was employed to test the effects of **21MO** on the MMP change. Upon addition of MPP^+^ the mitochondria of SH-SY5Y cells were depolarized (decrease of MMP) and notably **21MO** reversed the MMP change at 1 μM, but **17MN** did not, under the same experimental settings (Figure 3B). Combining the results from MC65 cells, this may indicate that **21MO** can reverse the changes induced by either production of AβOs in MC65 cells or MPP^+^ in SH-SY5Y cells on mitochondria, but **21MO** probably does not affect the MMP by itself.

**Figure 4:**
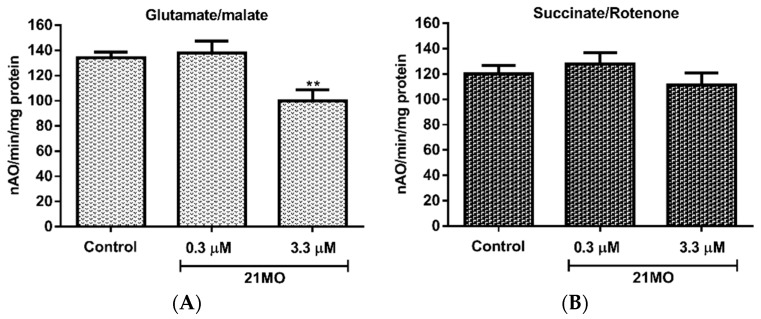
Effects of **21MO** on the oxy-phosphorylation of mouse brain mitochondria. (**A**,**B**) Oxygen consumption in mitochondria was measured using a Clark-type oxygen electrode at 30 °C using glutamate/malate (**A**) and using succinate/rotenone (**B**) in the presence or absence of **21MO** (** *p* < 0.01).

Taken together, the results might suggest that **17MN** and **21MO** interact differentially with mitochondria with **21MO** being more selective to complex I proteins, but these potential mitochondrial proteins all underlie the AβO-induced MMP change. To further confirm this notion and examine whether **17MN** and **21MO** interact with mitochondria differently, we tested **21MO** in the isolated brain mitochondria of mice for its effects on oxy-phosphorylation. Our previous studies have demonstrated that **17MN** does not show any significant effects on the oxy-phosphorylation of brain mitochondria [15]. As shown in Figure 4, notably, **21MO** significantly decreased the oxidation of glutamate (Figure 4A), the substrate of complex I, but not succinate, the substrate of complex II (Figure 4B), thus suggesting that **21MO** may preferentially target the complex I of brain mitochondria, which is consistent with results from the SH-SY5Y model.

**Figure 5:**
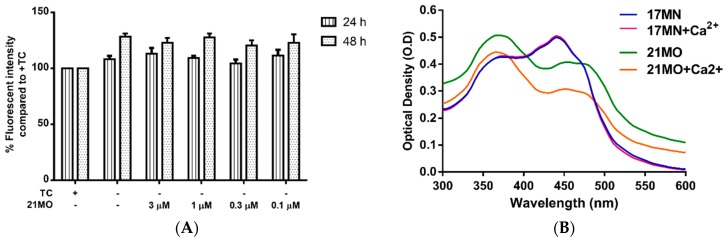
The impact of **21MO** on cytosolic Ca^2+^ levels in MC65 cells under −TC conditions. (**A**) MC65 cells were treated with **21MO** at indicated concentrations under −TC conditions for 24 and 48 h. Cells were then incubated with Fluo-4AM (2 µM) for 30 min. Mean fluorescence intensity was measured by flow cytometry; (**B**) Both **17MN** and **21MO** (50 µM) were incubated with CaCl_2_ (60 µM) at room temperature for 10 min, then the UV-vis spectrum was recorded from 300 nm to 600 nm.

Our previous studies demonstrated that withdrawal of TC resulted in a significant rise of intracellular Ca^2+^ levels in MC65 cells and the mobilization of Ca^2+^ originated from ER. Furthermore, **17MN** can efficiently suppress the increased Ca^2+^ level [15]. Therefore, we decided to compare the effects of **21MO** on Ca^2+^ levels upon TC-removal in MC65 cells to that of **17MN**. Surprisingly, **21MO** did not show any significant effects on the increased Ca^2+^ level at concentrations up to 3 μM (Figure 5A) after 24 and 48 h of TC removal. Our previous results have shown that **17MN** dose-dependently prevented this increase [15]. The time dependent change of Ca^2+^ upon TC removal is consistent with the production of AβOs [12], thus suggesting a role of Aβ in the calcium mobilization in this cell model. Further chelating experiments suggested that **21MO** does not a form complex with Ca^2+^ as evidenced by the unchanged maximum absorption (Figure 5B), similar to the results of **17MN**.

**Figure 6:**
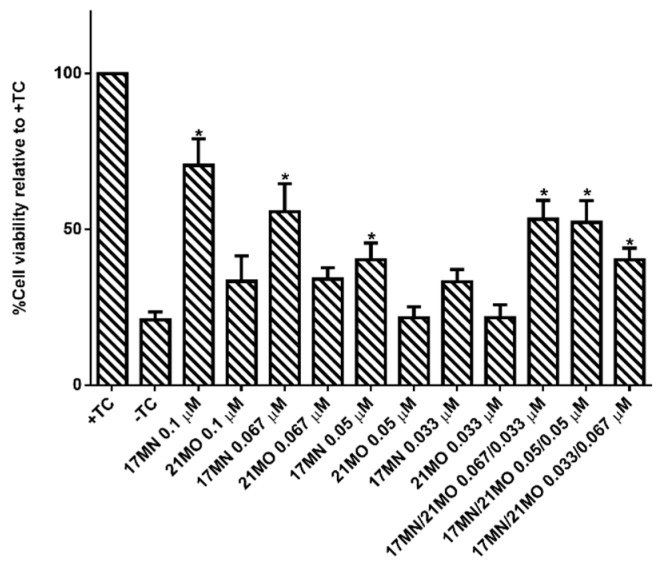
Protective activities of **17MN**, **21MO** or the combination of **17MN** and **21MO** on MC65 cells. MC65 cells were treated with indicated compounds at indicated concentration under −TC conditions for 72 h. Then cell viability was measured by MTT assay. Data were presented as mean (*n* = 4) ± SEM (* *p* < 0.05 compared to −TC).

Our previous studies suggested that **17MN** can readily pass into MC65 cells and localize into both mitochondria and ER, consistent with the observed effects of **17MN** on both MMP and Ca^2+^ change. The results of **21MO** in MC65 cells may suggest that this bivalent compound with a longer spacer may preferentially interact with mitochondria, but not ER, to exert its neuroprotective activities. The results also implicate that, upon the production of Aβ, especially AβOs, in MC65 cells, multiple pathways are potentially involved to elicit the observed cell death, consistent with the pathogenic complex nature of AD. Our previous studies observed the potency difference of **17MN** and **21MO** in protecting MC65 cells with **17MN** being more potent. This may echo the fact that **17MN** can interfere with both mitochondria and ER while **21MO** only preferentially interfere with mitochondria in MC65 cells, thus consistent with our original design rationale of developing compounds with multifunctions as effective treatments for AD. To further confirm this, we tested **17MN**, **21MO**, and the combination of these two for the protective activities under TC-removal conditions in MC65 cells. As shown in Figure 6, **17MN** rescued MC65 cells up to 71% survival at 0.1 μM while **21MO** can only slightly increase the survival rate of MC65 cells to 33% at the same concentration, consistent with our previous results [12,13]. When examining various combinations of **17MN** and **21MO** with the total concentration being 0.1 μM, the results showed that the observed protections from these combinations are comparable to that of **17MN** alone at corresponding concentrations. The results are consistent with the observed mechanistic study results and our hypothesis that the observed optimal protections of MC65 cells by **17MN** are probably due to its dual interactions with both mitochondria and ER, compared to **21MO**’s preferential interaction with only mitochondria.

## 3. Materials and Methods

### 3.1. Cell Lines and Reagents

MC65 cells (kindly provided by Dr. George M. Martin at the University of Washington, Seattle, WA, USA) were cultured in Dulbecco’s Modified Eagle’s Medium (DMEM) (Life Technologies, Inc., Grand Island, NY, USA) supplemented with 10% of heat-inactivated fetal bovine serum (FBS) (Hyclone, Logan, UT, USA), 1% Penicillin/Streptomycin (P/S) (Invitrogen, Carlsbad, CA, USA), 1 μg/mL Tetracycline (TC) (Sigma Aldrich, St. Louis, MO, USA), and 0.2 mg/mL G418 (Invitrogen). SH-SY5Y cells (ATCC) were cultured in DMEM supplemented with 10% FBS and 1% P/S. All cells were maintained at 37 °C in a fully humidified atmosphere containing 5% CO_2_.

### 3.2. MC65 Neuroprotection Assay

MC65 cells were washed twice with PBS, resuspended in Opti-MEM, and seeded in 96-well plates (4 × 10^4^ cells/well). Indicated compounds were then added, and cells were incubated at 37 °C under −TC conditions for 72 h. Then, 10 µL of MTT solution (3-(4,5-Dimethylthiazol-2-yl)-2,5-diphenyltetrazolium bromide 5 mg/mL in PBS) were added and the cells were incubated for another 4 h. Cell medium was then removed, and the remaining formazan crystals produced by the cellular reduction of MTT were dissolved in 100 µL of DMSO. Absorbance at 570 nm was immediately recorded using a FlexStation 3 plate reader (Molecular Devices, Sunnyvale, CA, USA). Values were expressed as a percentage relative to those obtained in the +TC controls.

### 3.3. U937 Necroptosis Assay

U937 cells (2 × 10^4^ cells) were pretreated with compound and caspase pan inhibitor Z-VAD for 1 h. TNF-α was then added and cells were incubated for 72 h. Afterward, 10 μL of MTT solution was added and cells were incubated for an additional 4 h. Cell medium was then removed, and the remaining formazan crystals produced by the cellular reduction of MTT were dissolved in 100 µL of DMSO. Absorbance at 570 nm was immediately recorded using a FlexStation 3 plate reader. Values were expressed as a percentage relative to those obtained in untreated controls.

### 3.4. Aβ_40_ ELISA

MC65 cells were collected and washed twice with PBS, resuspended in Opti-MEM, seeded in 6 well plates (1.6 × 10^6^ cells/well), and incubated with compounds at 37 °C under −TC conditions for 48 h. After centrifugation of the plates, 2 mL of medium was carefully collected for analysis of Aβ_40_ in medium. Cell pellets were lysed by cell extraction buffer (Life Technologies) following the manufacturer’s instructions and the total protein content was quantified by the Bradford method. Samples were analyzed using the Aβ_40_ Human ELISA Kit (Life Technologies) following the manufacturer’s instructions. The results were normalized by total protein expressed as a percentage relative to those obtained in the −TC control.

### 3.5. MC65 Mitochondrial Membrane Potential Assay

MC65 cells were collected and washed twice with PBS, resuspended in Opti-MEM, seeded in 6 well plates (1.6 × 10^6^ cells/well), and incubated with compounds at 37 °C under −TC conditions for 48 h. Cells were then collected, washed twice with PBS, and then incubated with 100 nM of tetramethylrhodamine methyl ester (TMRM) in PBS at room temperature for 30 min. Fluorescence was analyzed by flow cytometry.

### 3.6. SH-SY5Y Mitochondrial Membrane Potential Assay

SH-SY5Y cells (4 × 10^5^ cells) were plated in 12 well plates. After incubation for 24 h, growth medium was removed and cells were treated in DMEM with indicated compounds and MPP^+^(2.5 mM) for 24 h. TMRM was then added to a final concentration of 100 nM and the cells were further incubated for 30 min. The medium was then collected. The cells were detached by trypsinization, and the medium was then recombined with the cells and centrifuged. After removing the supernatant, the cell pellet was suspended in PBS and the mean fluorescent intensity was recorded by flow cytometry.

### 3.7. Brain Mitochondrial Isolation and Functional Determination

The Institutional Animal Care and Use Committees (IACUC) of the McGuire VA Medical Center and Virginia Commonwealth University approved this protocol. Brain cortex tissue was collected from C57BL/6 mice after the mouse was anesthetized with sodium pentobarbital (100 mg/kg i.p.) and the heart was removed. Harvested brain tissue was placed into 5 mL MSM buffer (210 mM Mannitol, 70 mM Sucrose, 5.0 mM MOPS, 1.0 mm EDTA, pH 7.4) at 4 °C, and finely minced and incubated with Subtilisin A (1 mg/g tissue) for 1 min. Another 5 mL MSM buffer including 0.2% BSA was added to incubated tissue that was then homogenized by one stroke using a Teflon pestle. The homogenate was centrifuged at 600 g for 10 min at 4 °C. The supernatant was then centrifuged at 5000 *g* for 10 min at 4 °C to spin down the mitochondria. The mitochondrial pellet was washed once with MSM buffer, then re-suspended in 100–200 µL of MSM buffer. Total protein concentration was measured by the Lowry method using BSA as a standard. Oxygen consumption in mitochondria was measured by a Clark-type oxygen electrode at 30 °C using glutamate + malate (complex I substrates) and succinate + rotenone (complex II substrates) in the presence or absence of compound **17MN** [18].

### 3.8. MC65 Calcium Level Measurement

MC65 cells were washed twice with PBS, resuspended in Opti-MEM, and seeded in 6-well plates (1.6 × 10^6^ cells/well). Indicated compounds were then added, and cells were incubated at 37 °C under −TC conditions for 48 h. Cells were then harvested, suspended in PBS, and incubated with Fluo-4AM (2 µM) in dark for 30 min. Cells were washed once and then resuspended in PBS. Samples were analyzed by flow cytometry. Values were expressed as a percentage relative to those obtained in −TC controls.

### 3.9. Biometal Complex Assay

Compound **17MN** and **21MO** (50 µM) were incubated along with CaCl_2_ (60 µM) in water (100 µL) were incubated at room temperature for 10 min, then UV absorption was recorded from 300 nm to 600 nm using a FlexStation 3 plate reader.

### 3.10. Statistical Analyses

The Student’s *t*-test was used for all statistical analysis. Data are presented as mean ± SEM. The level of significance for all analysis testing was set at * *p* < 0.05.

## 4. Conclusions

In summary, our studies suggested that bivalent compound **21MO** can protect MC65 cells from TC-removal induced necroptosis, probably via interactions with the complex I of mitochondria. But **21MO** did not have any impact on the Ca^2+^ change under the current experimental settings. The results collectively suggest that **17MN** and **21MO** may have different mechanisms of action for their neuroprotective activities, even though they share the same overall structure with both curcumin and a cell membrane anchor moieties linked by a spacer. This also suggests that the cell membrane anchor and the spacer do play an important role in their biological activities, consistent with our original design rationale and reported results. Further studies are being undertaken to evaluate specifically the impact of spacer or cell membrane anchor on their biological activities by employing newly designed bivalent probes.
